# Wide-field cellular-resolution retinal imaging using deformable mirror-based sensorless adaptive optics time-domain full-field OCT

**DOI:** 10.1364/BOE.579540

**Published:** 2025-11-17

**Authors:** Yao Cai, Olivier Martinache, Maxime Bertrand, Clémentine Callet, Olivier Thouvenin, Kate Grieve, Pedro Mecê

**Affiliations:** 1 Institut Langevin, ESPCI Paris, CNRS, PSL University, Paris, France; 2 Sorbonne Université, INSERM, CNRS, Institut de la Vision, Paris, France; 3 SharpEye SAS, Gentilly, France

## Abstract

Adaptive optics (AO) enables cellular-resolution retinal imaging by correcting ocular aberrations, but its widespread clinical adoption remains limited by the narrow field of view (FOV) imposed by the isoplanatic patch of the eye. In this study, we present a deformable mirror (DM)–based sensorless AO time-domain full-field OCT (FFOCT) system that overcomes these limitations by leveraging the inherent robustness of FFOCT to ocular aberrations under spatially incoherent illumination. Using both phantom eye simulations and in vivo experiments, we demonstrate that correction of only three to five Zernike modes (defocus, astigmatism, and coma) is sufficient to significantly enhance SNR and resolve fine retinal structures. This includes reliable visualization of cone photoreceptors as close as 0.3^∘^ from the foveal center and depth-resolved imaging of inner retinal features such as nerve fiber bundles, vessel walls, capillaries, internal limiting membrane, macrophage-like cells, and Gunn’s dots, across a $5^{\circ }\times 5^{\circ }$ FOV at 500 Hz. By simplifying AO implementation while achieving wide-field cellular resolution, this approach addresses key limitations of current AO ophthalmoscopes and offers a promising pathway toward a wider clinical deployment of high-resolution retinal imaging.

## 1. Introduction

Cellular-resolution retinal imaging is a crucial tool for the early diagnosis of retinal diseases, monitoring disease progression, and evaluating emerging therapies [1,2]. Achieving such fine details requires adaptive optics (AO), a technology that corrects ocular aberrations in real time to achieve a diffraction-limited resolution [3]. AO has significantly improved various retinal imaging modalities, including flood illumination ophthalmoscopy (FIO) [3,4], scanning light ophthalmoscopy (SLO) [5,6], and optical coherence tomography (OCT) [7,8]. By allowing high-resolution imaging through a dilated pupil, these techniques allow visualization of critical microstructures such as individual photoreceptors and nerve fiber bundles, thereby offering unprecedented insights into retinal morphology and function [2,9].

Despite these advances, AO retinal imaging typically suffers from a narrow field of view (FOV), which can be explained by two main factors. First, most AO imagers are based on point-scanning illumination/detection (e.g., AO-SLO and AO-OCT), which imposes a trade-off between spatial sampling and eye motion to avoid motion-induced artifacts. Second, the isoplanatic patch of the eye (the area in which AO correction is effective) is limited to around $1^{\circ }–2^{\circ }$ due to field-dependent aberrations [10]. Consequently, multiple images must be acquired to cover a larger retinal area, which is time consuming and impractical in clinical workflows. To mitigate this, some groups have reduced the numerical aperture to enlarge the isoplanatic patch [11–15], at the cost of lateral resolution. Multi-conjugate AO has also been explored [16,17], although at the expense of significant system complexity.

To address these challenges, our group recently introduced an alternative approach that combines sensorless AO with time-domain full-field OCT (FFOCT) for high-resolution retinal imaging over a larger FOV in a compact design [18,19]. We demonstrated that with spatially incoherent illumination, the lateral resolution of FFOCT is less affected by low-order symmetric aberrations [20,21], which dominate static, dynamic, and field ocular aberrations [10,22]. Therefore, by using a multi-actuator adaptive lens (MAL) placed directly in front of the eye without strict pupil conjugation, combined with sensorless wavefront optimization, we were able to image photoreceptors as close as 0.5^∘^ from the foveal center across a $5^{\circ }\times 5^{\circ }$ FOV [18,19]. However, MAL performance was limited by three main factors: (1) insufficient stroke relative to the amplitude of low-order ocular aberrations, (2) hysteresis, and (3) the inability to use Zernike-based optimization due to hysteresis. As a result, the system was unable to consistently achieve cellular resolution or maintain a high signal-to-noise ratio (SNR), particularly for inner retinal imaging and when imaging subjects with high refractive errors.

In this manuscript, to overcome the limitations of the multi-actuator adaptive lens, we propose a novel AO sensorless time-domain FFOCT design and system, featuring a pupil-conjugated deformable mirror (DM). The high stroke of the DM and negligible hysteresis allow accurate coherence gate and focal plane matching, more precise and repeatable wavefront correction, and enable Zernike mode–based optimization. Our results, validated through both phantom eye simulations and in vivo experiments, demonstrate that correcting only three to five Zernike modes is sufficient to achieve high SNR and resolve fine retinal structures, including foveal cones and inner retinal features, across a $5^{\circ }\times 5^{\circ }$ FOV. Furthermore, we propose a method to investigate alternative sensorless AO designs to enhance the robustness of FFOCT imaging performance across different retinal layers and among subjects.

## 2. Methods

### 2.1. FFOCT imaging system design

**Fig. 1.**
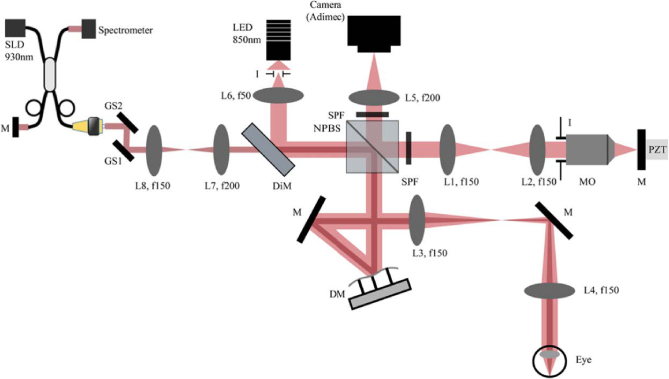
Optical layout of the DM-based sensorless AO FFOCT. The light red path corresponds to the time domain FFOCT module, while the dark red path outlines the optical path of the SD-OCT. L: lens, NPBS: non-polarizing beam-splitter, I: iris, SPF: short-pass filter, M: mirror, DM: deformable mirror, MO: microscope objective, PZT: piezoelectric transducer, GS: galvanometer scanners, SLD: superluminescent diode, LED: light-emitting diode.

Figure 1 presents a schematic of the custom-built DM-based sensorless AO FFOCT system. The FFOCT system comprises a spatially incoherent light-emitting diode (LED) with a center wavelength of 850 nm and a bandwidth of 30 nm (M850L3, Thorlabs), providing a theoretical axial resolution of approximately 8 *μm* m in water. The LED is focused by a condenser (focal length: 50 mm) positioned in front of the system’s pupil plane, implementing a Köhler illumination configuration. A field diaphragm in front of the LED is optically conjugated to both the retina and the reference mirror. A 50:50 cubic non-polarizing beam splitter (BS) divides the illumination beam into the reference and sample arms. In the reference arm, a pair of achromatic doublets (focal length: 150 mm) direct light toward a microscope objective (Olympus 10X/0.25 NA plan Achromat), which focuses the beam onto a silicon reference mirror. Both the microscope objective and the reference mirror are mounted on a high-speed voice-coil translation stage (X-DMQ12P-DE52, Zaber Technologies Inc.), enabling rapid optical path length adjustments in the refenrence arm. In the sample arm, a deformable mirror (DM) with a clear aperture of 7.2 mm (DM97-08, Alpao, France) is relayed to the eye’s pupil with 1:1 magnification using a pair of achromatic doublets (focal length: 150 mm). The aperture of the system is ultimately limited by the 7.2 mm DM size. To compensate for dispersion effects and curvature mismatch between the FFOCT coherence gate and the retina, an optical window is introduced in the sample arm between L4 and the eye (not shown in Fig. 1) [23]. Photons reflected from the reference mirror and backscattered from the sample recombine at the BS and are focused by an achromatic doublet (focal length: 200 mm) onto a high-speed 2D megapixel (1440 × 1440 pixel) camera (Q-2A750-Hm/CXP-6, Adimec), with 1 *μm*/pixel retinal sampling.

A spectral-domain optical coherence tomography (SD-OCT) system (GAN611, Thorlabs) is integrated into the setup. The SD-OCT system utilizes a broadband superluminescent diode with a center wavelength of 930 nm and a 60 nm bandwidth, resulting in a theoretical axial resolution of 4.5 *μm* in water. Unlike our previous setup [18], the pupil plane of the system is now optically conjugated to the midpoint between both galvanometer scanners of the SD-OCT, reducing pupil wobble [24], and enhancing the quality of the B-scan, and field of view. This conjugation is achieved using a pair of achromatic doublets (focal lengths: 150 mm and 200 mm, respectively). A long-pass dichroic mirror couples the SD-OCT to the FFOCT optical path. To compensate for dispersion in the SD-OCT, additional optical windows are incorporated into the reference arm of the SD-OCT, while two short-pass filters are used to block the SD-OCT light in both the reference and detection arms of the FFOCT. The numerical aperture of the SD-OCT is also limited by the DM’s 7.2 mm clear aperture.

B-scan measurements are performed at a 100 kHz A-scan rate with 256 scanning points on a 1^∘^ field of view. Each acquired B-scan is laterally averaged and serves three key purposes: 1) measuring the retinal axial position to minimize optical path length differences between the reference and sample arms in FFOCT, allowing for real-time retinal axial motion correction [25,26]; 2) providing a real-time cross-sectional view for precise positioning of the FFOCT coherence gate at a specific retinal layer of interest; 3) acting as a merit function for sensorless wavefront optimization by maximizing the brightness of a selected retinal layer.

### 2.2. Sensorless adaptive optics design

**Merit function:** Although the brightness of the FFOCT signal is affected by ocular aberrations, it cannot be used as a reliable merit function due to intensity fluctuations also arising from inaccurate two-phase modulation induced by axial retinal motion [26]. To circumvent this issue, we used the brightness of the SD-OCT B-scan as a surrogate for FFOCT SNR optimization. Given the close wavelengths of both systems, wavefront optimization using SD-OCT brightness as the merit function effectively enhances the FFOCT SNR as well (see Supplement 1 Figure S1).

**Algorithms for wavefront optimization:** In this study, two algorithms, specifically optimized for in vivo retinal imaging, with rapid convergence and robust optimization, were investigated: the Hill Climbing (HCl) algorithm proposed by Camino *et al.* [15] and the Data-based Online Nonlinear Extremum Seeker (DONE) algorithm introduced by Verstraete *et al.*[27,28].

• **Hill Climbing (HCl) algorithm** optimizes the merit function mode by mode, using adaptive step sizes to find the optimized Zernike coefficient for each mode. The step size is updated on the basis of the gradient sign of the merit function from the previous iteration. Empirical validation in a phantom eye yielded the selection of the following parameters: an initial step size of 0.3 *μ*m, a maximum step size limit of 2.5 *μ*m, 9 iterations per mode, an ϵ parameter of 0.3 *μ*m to prevent local minima trapping, and 3 optimization cycles to mitigate mode coupling [29].
• **DONE algorithm:** Unlike HCl, the DONE algorithm is a model-based approach that simultaneously explores all the targeted Zernike modes. It is highly robust to noisy data, as it incorporates all previous measurements to update the model at each iteration. The unknown merit function is modeled using a Random Fourier Expansion (RFE), which is fitted to the experimental data via a least-squares approach. The optimization iteratively minimizes the merit function by updating the RFE with each new measurement and uses the updated RFE as a surrogate merit function for subsequent measurements. The search range for Zernike coefficients was selected based on the root-mean-square (RMS) wavefront error measurements from an ocular aberration dataset [22]: Defocus: -2 to 2 *μ*m RMS; Astigmatism: -1.5 to 1.5 *μ*m RMS; Remaining Zernike modes (up to spherical aberration): -1 to 1 *μ*m RMS. For subjects whose defocus exceeded this predefined range, an initial manual defocus adjustment was performed, after which the optimization was carried out within the same range around the manually optimized defocus value. Hyperparameters were chosen based on our previous in vivo retinal imaging work [18]. The number of basis functions, *D*, was set to 200; increasing *D* improves the RFE fit, but slows the computation. To balance under- and overfitting, a regularization parameter λ=0.01 was used in the least-squares fit for RFE coefficient estimation, which also helps when dealing with limited measurements. The probability density function for the RFE model follows a Gaussian distribution defined by two parameters: σ=1 , which controls the variance of the random Fourier expansion frequencies, and ϵ=0.02 , which influences RFE surrogate exploration. Increasing ϵ can be useful if the algorithm gets trapped in local minima but potentially reducing precision near the optimum [18].

**Wavefront optimization duration:** Given that the average tear-film breakup time in normal subjects and those with moderate dry eye is approximately 4 s and 2 s, respectively, our goal was to design both the HCL and DONE algorithms so that the wavefront optimization duration remained below 2 s, in order to accommodate tear-film dynamics [30]. For the HCL algorithm, the optimization duration is determined by several parameters: the computation time per iteration (2 ms), the effective DM settling time (7 ms) per iteration, the number of iterations per mode (9 iterations), the number of corrected modes, and the number of optimization cycles (3 cycles). For instance, when correcting 8, 5, and 3 Zernike modes using the HCL algorithm, the corresponding optimization durations are 2 s, 1.2 s, and 0.7 s, respectively. In contrast, the DONE algorithm optimizes all Zernike modes simultaneously, resulting in a longer computation time per iteration (26 ms). Given 50 iterations and considering the effective DM settling time, the total optimization duration is approximately 1.65 s.

It is worth noting that for a small number of modes, the optimization duration was sufficient to converge towards a stable maximum of the SD-OCT signal intensity (merit function). However, this was not always the case when optimizing 5 or 8 modes, where additional correction cycles (for HCL) or iterations (for DONE) would be necessary. Since dynamic ocular aberrations evolve over a timescale of only a few seconds, extending the optimization beyond approximately 2 s would not necessarily improve convergence and could even reduce performance. Therefore, we limited the maximum optimization duration to 2 s.

**Optimization of the DM response:** An important consideration for efficient sensorless AO is to ensure that the merit function is not affected by DM overshoots or oscillations during optimization. Most DMs, such as the Alpao DM97-08 used in this study, are designed for closed-loop operation with small step sizes to correct residual aberrations, achieving a typical settling time of 0.62 ms to within ±10% of the command value. However, the sensorless approach often requires larger step sizes (e.g., 2 μm), which can lead to transient oscillations of the mirror surface before full stabilization. These oscillations introduce fluctuations in the merit function (SD-OCT signal intensity) that are unrelated to aberration correction, thereby reducing the accuracy and repeatability of the optimization.

To minimize this effect while maintaining a fast response, the DM slew rate was adjusted. A higher slew rate reduces oscillations but increases response time, whereas a lower slew rate shortens the settling time at the cost of larger overshoots. In this study, we selected a slew rate of 25 steps, which provided a good compromise between speed and stability, achieving rapid DM stabilization ( 7 ms) with negligible oscillations.

**Protocol to investigate the optimal sensorless AO design:** To investigate the number of Zernike modes that should be corrected in time-domain FFOCT retinal imaging, we used a phantom eye previously described in [21]. In summary, we placed the same microscope objective in the sample arm as in the reference arm, ensuring symmetry between both arms, and a lens-cleaning tissue was positioned at the focal place of the microscope objective as the imaging sample, to mimic the scattering behavior of the retina. To simulate the effect of static ocular aberrations, we used the 50 eye ocular aberration database collected by Jarosz *et al.* [22]. This database provides high spatiotemporal resolution ocular aberrations up to the 8th Zernike radial order, collected at 236 Hz over a period of 3.5 s. Initially, the database was recorded for a 5-mm pupil size. In this study, to simulate aberrations at a pupil diameter of 7.2 mm (the typical aperture for high-resolution retinal imaging), we converted the Zernike coefficients using the method described by Campbell [31]. The converted Zernike coefficients were consistent with data from the literature on static aberrations for pupils around 7 mm [32].

To assess the effect of sensorless AO on different levels of aberrations, we classified the 50 eyes into three groups based on their high-order aberration (HOA) wavefront error, excluding defocus and astigmatism. The HOA wavefront error was defined as $WFE_{HOA}=\sqrt{\sum c_{k}^{2}}$ , where *c* are the Zernike coefficients of HOA (excluding piston, tip/tilt, defocus and astigmatism). These groups were: 1) Group 1 (10 eyes with the weakest HOA), 2) Group 2 (10 eyes with mid HOA), and 3) Group 3 (10 eyes with the strongest HOA). For each group, we randomly selected five subjects, ensuring that their overall aberrations were representative of the entire group.

For each subject, we applied the temporally averaged Zernike coefficients of the first 38 modes (excluding piston, tip, and tilt) to the DM to simulate static ocular aberrations. We then executed both sensorless AO algorithms (HCl and DONE) to optimize different numbers of Zernike modes: 1, 3, 5, and 8 modes (starting from defocus). For each subject aberrations, FFOCT images and SDOCT B-scans were acquired under six conditions and repeated five times: 1)under static ocular aberrations to simulate photoreceptor layer (PRL) imaging (no sensorless AO correction), 2) only defocus optimized (1 mode), 3) defocus and astigmatisms optimized (3 modes), 4) first 5 modes (up to coma) optimized, 5) first 8 modes (up to spherical aberration) optimized, and 6) at the diffraction limit (no aberration added, with the DM correcting for residual system aberrations). Furthermore, nerve fiber layer (NFL) imaging was simulated by adding 1.5 *μ*m RMS defocus (equivalent to 0.5 D) to the static ocular aberrations.

For each subject and sensorless AO correction condition, 50 FFOCT images were acquired at 500 Hz using a 2-phase modulation step consistent with in vivo retinal imaging. The 2-phase step was implemented by modulating the reference mirror of the FFOCT with a piezoelectric transducer (PZT, ENT400, TRIOPTICS France). To analyze the FFOCT signal, we first minimized the effect of phase instability and signal fluctuation by averaging only the five brightest en-face images (where the 2-phase modulation is the most precise) out of the 50 acquired images. The signal level was then computed as the mean value of the 10,000 brightest pixel values, excluding areas without signal. The noise level was computed as the standard deviation of a region of interest (100 × 100 pixels) where no signal was present. Finally, the SNR was calculated as the ratio of the signal level to the noise level.

### 2.3. In-vivo image acquisition and processing

In vivo retinal imaging was performed in six healthy subjects (aged 25 to 35), with a spherical equivalent refraction between 0 and -4.5 diopters. The research procedures adhered to the principles outlined in the Declaration of Helsinki. Informed consent was obtained from all subjects after explaining the nature of the study and potential outcomes. The study was approved by the relevant ethics review boards [CPP and ANSM (IDRCB number: 2019-A00942-55)]. All imaging sequences were acquired in a dark room. The subjects’ eyes were cyclopleged and dilated using 0.5% tropicamide. Each subject was seated in front of the system and stabilized with a chin and forehead rest mounted on a 3D translation stage and instructed to fixate on a target. For some subjects, an initial manual defocus compensation was performed prior to starting sensorless AO correction. This step increased the brightness of the targeted retinal layer, which was used as the merit function, thereby enhancing the accuracy and reproducibility of the wavefront optimization. Images were captured immediately after wavefront optimization and during real-time correction of axial eye motion. Each image sequence comprised 500 frames of 1440 × 1440 pixels (FOV: $5^{\circ }\times 5^{\circ }$ ) acquired within 1 s. For five subjects, only defocus and astigmatism (3 Zernike modes) were optimized. For one subject who had undergone Lasik surgery, which is known to increase high-order aberrations [33], wavefront optimization included the first 5 Zernike modes. During image acquisition, the total optical power entering the eye was 1.3 mW from the FFOCT illumination (for the 1-s acquisition) and 0.25 mW from the SD-OCT (for continuous scanning), respectively, which are below the ocular safety limits established by ISO standards for group 1 devices. Image processing steps, previously detailed in [25], included: 1) image normalization by dividing each frame by its mean intensity; 2) 2-phase demodulation by subtracting consecutive images and computing their absolute value; 3) image selection to exclude frames acquired during blinks, micro-saccades, or those with low SNR due to poor phase modulation; 4) image registration to correct both translation and rotation using a phase correlation method described in [34]; and 5) image averaging.

## 3. Results

### 3.1. Optimizing sensorless AO correction in FFOCT

**Fig. 2.**
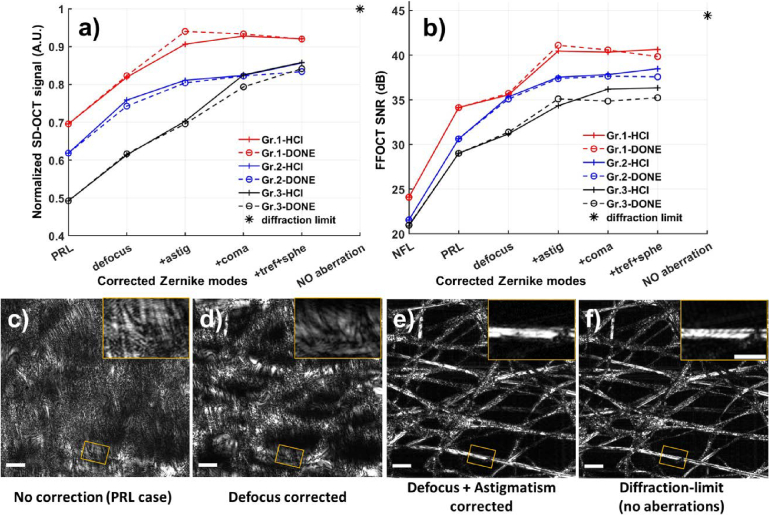
(a) Averaged SD-OCT signal intensity as a function of corrected Zernike modes. SD-OCT averaged signal was normalized by the diffraction-limit value. b) Averaged FFOCT SNR as a function of corrected Zernike modes. Averaged values were computed over 25 measurements (5 subjects × 5 repetitions) for each HOA group. Seven conditions were studied, NFL: no aberration correction + additional 1.5 *μ*m RMS defocus; PRL: no aberration correction; 3) defocus optimized; 4) defocus + astigmatism (3 modes) optimized; 5) up to come (5 modes) optimized ; 6) up to spherical aberration (8 modes) optimized; 7) no aberration (diffraction-limit case). (c-f) Normalized FFOCT images of lens cleaning tissue under different aberration correction conditions: c) all aberrations present, d) defocus corrected, e) defocus + astigmatism corrected, f) no aberration (diffraction-limit case). Wavefront optimization was performed using the HCl algorithm. Magnified images are presented in the upper right corner of each image. Scale bar: 50 *μ*m and 30 *μ*m for the FFOCT images and the magnified images respectively.

To study the optimal number of Zernike modes to be corrected, we used the phantom-eye protocol previously described. Figure 2 (a,b) shows the average signal level (SNR) for SD-OCT and FFOCT as a function of the number of optimized Zernike modes, using DONE or HCl algorithms. The SD-OCT signal, used as the merit function, demonstrates that both the DONE and the HCl algorithms produce similar performance and convergence trends (Fig. 2(a)). Groups 1 and 2 achieved optimal signal enhancement when correcting up to astigmatism, reaching 90% and 80% of the signal strength compared to the non-aberration condition, while the correction for coma and spherical aberration provided minor additional gains. In contrast, for group 3, correcting at least up to coma yields optimal performance, reaching the same signal recovery (80% of diffraction-limited) that group 2 attained with correction up to astigmatism.

Given that the SD-OCT and FFOCT illumination wavelengths are close, the SD-OCT signal intensity can serve as a surrogate merit function for FFOCT. As shown in Fig. 2(b), the evolution of the FFOCT signal closely mirrors that of the SD-OCT signal. For both DONE and HCL algorithms, the FFOCT signal shows comparable optimization performance, with HCL converging slightly faster for a small number of corrected modes and DONE exhibiting smoother convergence when optimizing a larger number of modes. Notably, when the wavefront is optimized up to astigmatism, the signal improvement saturates. Increasing the number of corrected modes yields negligible additional improvement, except for Group 3, which shows an extra gain of approximately 3 dB with the HCL algorithm.

For the NFL imaging case, correction up to astigmatism results in a significant increase in the FFOCT SNR (13-17 dB) in the three HOA groups and an improvement in the SNR of 5-7 dB for photoreceptor imaging. Compared to the diffraction limit (45 dB with no aberration), the sensorless AO method correcting for 3 modes (defocus and astigmatism) achieves 40 dB, 37 dB and 35 dB FFOCT SNR for groups 1, 2, and 3, respectively. These results are consistent with expectations, as group 3 presents the strongest high-order aberrations that are not corrected for in the sensorless AO optimization.

Figure 2(c-f) also includes FFOCT images of lens cleaning tissue acquired from group 1, illustrating that wavefront optimization up to astigmatism not only optimizes SNR but also results in high-resolution images that approach the diffraction-limit case.

### 3.2. In-vivo validation of optimal number of corrected modes

**Fig. 3.**
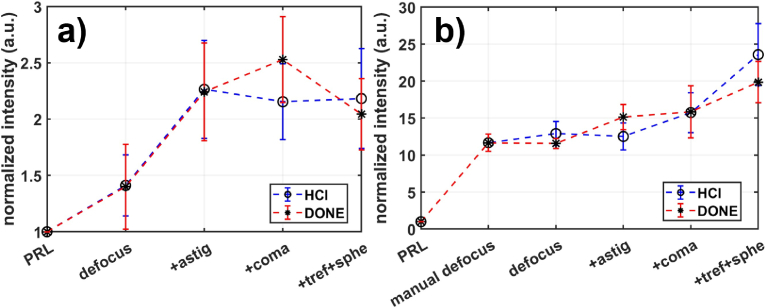
SD-OCT signal intensity under various aberration correction conditions in in-vivo retinal imaging of a) a healthy subject with -0.5 D myopia, and b) a healthy subject with a history of Lasik surgery. SD-OCT signal was normalized by the PRL signal level (no aberration correction). Note that the signal acquired from Subject 2 at PRL is very low, necessitating a manual defocus adjustment to attain a suitable signal level to start the sensorless AO optimization. The blue and red traces represent the signal intensity following the optimization with the HCl and DONE algorithms, respectively. Symbols and error bars denote the averaged value and the standard deviation of 5 repeated measurements under the same condition.

To verify that the findings from the static phantom eye are consistent with sensorless AO performance in vivo, we applied the same protocol using SD-OCT for two different subjects representing respectively HOA Group 1 and 3. Subject 1, with -0.5 D myopia, and Subject 2, with a history of Lasik surgery leading to increased high-order aberrations [33]. For each aberration correction condition (number of optimized modes), five repeated measurements were collected. Figure 3 shows the normalized intensity (mean ± SD) versus the number of optimized modes for both subjects. The SD-OCT signal was normalized by the single intensity of the PRL imaging without aberration correction.

For both subjects, the performance of the DONE and HCl algorithms was similar with minor differences observed: DONE performed slightly better when correcting up to coma for Subject 1 and up to astigmatism for Subject 2, while HCl performed slightly better when correcting up to spherical aberration for both subjects. For Subject 1 (Fig. 3(a)), after optimizing for 3 modes, the signal increased by a factor of 2.3. This improvement was slightly further enhanced to 2.5 when the DONE algorithm was used to correct up to coma (5 modes). The optimization trend for Subject 1 aligns with the behavior observed in groups 1 and 2 (Fig. 2(a)), with optimal correction achieved with 3-mode correction. Subject 2 (Fig. 3 (b)) exhibited a significant signal enhancement when correcting for 8 Zernike modes, consistent with the optimization trend in group 3 with a stronger HOA (Fig. 2(a)). While SD-OCT showed progressive signal increase, by factors of 13–15 with 3-mode correction and 20–24 with 8-mode correction, this improvement did not always translate into a significant gain for the FFOCT signal, which seemed to saturate after 5-mode correction (Fig. 2(b)).

Given that the evolution of the SD-OCT signal in vivo with different numbers of corrected Zernike modes follows the same optimization behavior as observed in phantom eye simulations, we can conclude that the optimal sensorless AO design for FFOCT in in vivo retinal imaging involves correcting 3 modes (defocus and astigmatism) for subjects in groups 1 and 2 (weak to mid high-order aberrations) and 5 modes (up to coma) for subjects in group 3 (strong high-order aberrations).

### 3.3. Cellular-resolution in-vivo retinal imaging

#### 3.3.1. Large field-of-view foveal photoreceptors imaging with enhanced resolution

**Fig. 4.**
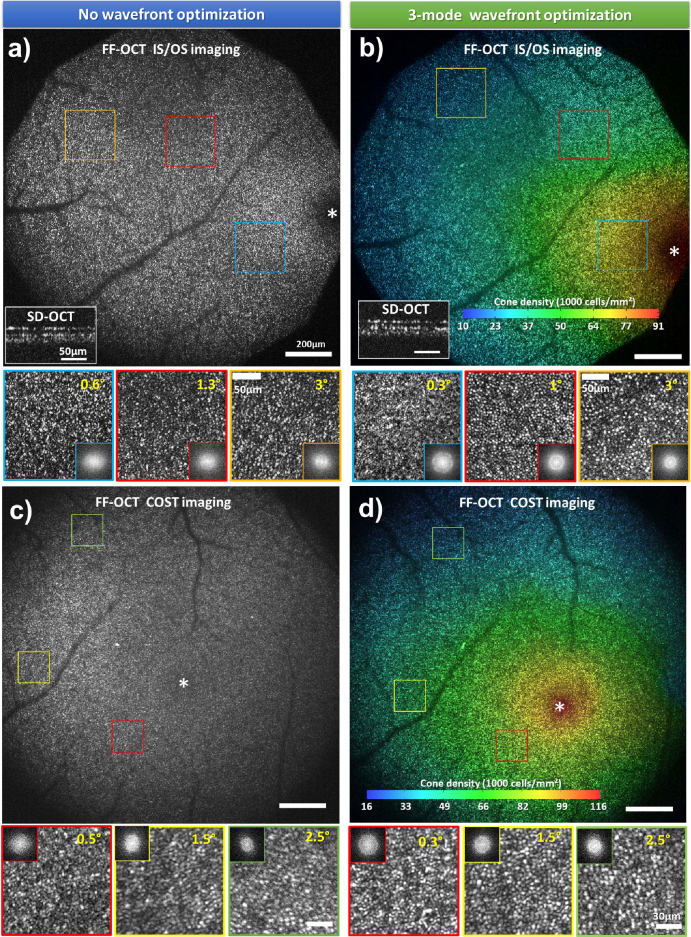
FFOCT retinal images of photoreceptors at the foveal center. (a,b) IS/OS images acquired before (a) and after (b) 3-mode wavefront optimization using DONE algorithm. (c,d) COST images acquired before (c) and after (d) 3-mode correction using DONE algorithm. SD-OCT cropped B-scans from the photoreceptor layer before and after wavefront optimization are presented on the left bottom corner of (a) and (b) respectively. Magnified images and corresponding Fourier transforms are shown below each image,demonstrating the resolution capacity through the well-defined Yellot’s ring, except at 0.5^∘^ and 0.6^∘^ eccentricity before wavefront optimization (a,c). IS/OS and COST images after wavefront optimization (b,d) are color-coded to represent the computed cone density (see colorbar).

In the SD-OCT channel, the insets in the bottom left corners in Figs. 4 a) and b) show cropped single B-scans before and after wavefront optimization, illustrating improvements in both SNR and resolution. Previously unresolved cone photoreceptors become clearly distinguishable as distinct, hyper-reflective dots at the inner/outer segment (IS/OS) junction and the cone outer segment tip (COST). The SNR enhancement facilitates more precise real-time axial eye tracking and a more stable merit function for sensorless AO. Visualization 1 provides an example of wavefront optimization using the SD-OCT B-scan, where the real-time evolution of the B-scan, its lateral average, and the merit function can be tracked throughout the optimization process.

FFOCT imaging also benefits from the 3-mode wavefront optimization using SD-OCT B-scans as the merit function. Figure 4 presents FFOCT images of foveal photoreceptors from the same subject, acquired before and after wavefront optimization. Real-time axial eye tracking allows precise positioning of the 8*μm* FFOCT coherence gate at specific retinal layers, enabling enface imaging of IS/OS and COST layers separately over a large FOV of $5^{\circ }\times 5^{\circ }$ , as shown in Figs. 4 (a,b) and (c,d), respectively.

After wavefront optimization, the SNR of the FFOCT images of IS/OS and COST increased by a factor of 2.3 and 2.8, respectively. Without aberration correction, cones could be resolved down to 1.5^∘^, demonstrating the robustness of time-domain FFOCT lateral resolution in the presence of ocular aberrations [20,21].Correcting defocus and astigmatism improved the FFOCT lateral resolution, enabling visualization of cones as close as 0.3^∘^ from the fovea, with distinct Yellot’s rings in the zoomed views (Fig. 4). This represents an improvement over our previous adaptive-lens sensorless AO approach, where cones were visible up to 0.5^∘^ from the foveal center [18,19].

In addition to the enhanced SNR and resolution, 3-mode wavefront optimization enabled clear cone visualization across the whole $5^{\circ }\times 5^{\circ }$ FOV, without any apparent anisoplanatism. Photoreceptor density was computed over the entire FOV (color-coded in Fig. 4 (b,d)) by analyzing the Fourier transform, using the method described in [18,35]. The wide FOV allowed for the measurement of the cone density ranging from 16,000 cones/mm^2^ at 3^∘^ eccentricity to 116,000 cones/mm^2^ near the foveal center, consistent with previously reported values [36]. Supplement 1 Figure S2 showcases photoreceptor imaging from different eccentricities and subjects, further highlighting the robustness of the applied wavefront optimization method combined with FFOCT to achieve cellular-resolution retinal imaging over a large FOV.

#### 3.3.2. Depth-resolved wavefront optimization for outer and inner retina imaging

Although the FFOCT coherence gate can be precisely positioned in the retinal layer of interest, a mismatch between the coherence gate and the focal plane (defocus aberration) reduces the SNR of FFOCT. At full aperture, with a 7 mm pupil diameter, the depth of focus is approximately ten times thinner than the retina, which makes precise focus critical [37,38]. If the merit function does not account for the specific retinal layer of interest, wavefront optimization becomes biased towards the photoreceptor layer [15], preventing high-SNR FFOCT imaging of the inner retina.

**Fig. 5.**
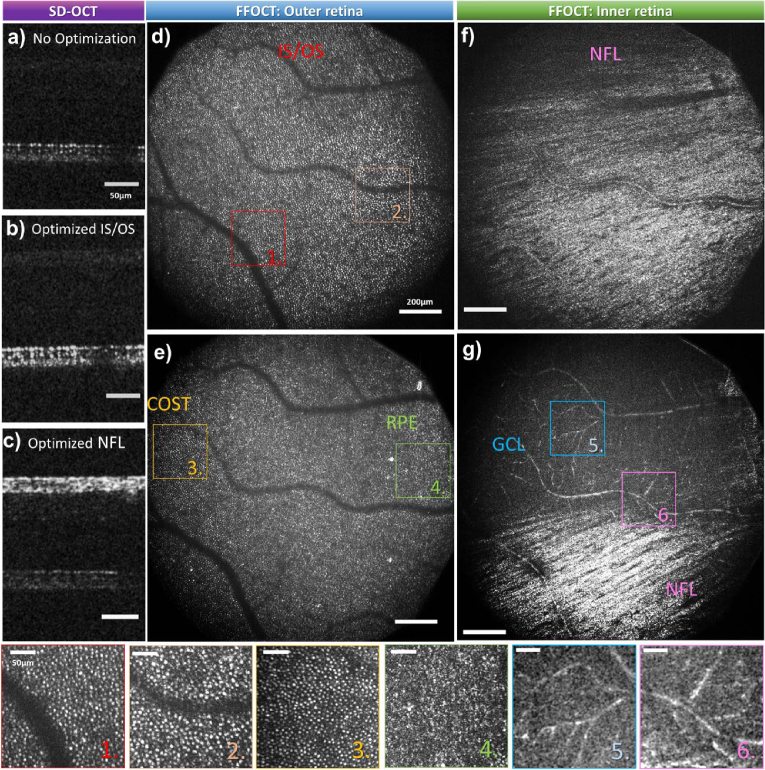
(a-c) SDOCT Bscans acquired before (a), after wavefront optimization for the IS/OS layer (b) and NFL (c). FFOCT en-face images acquired at the IS/OS (d) and COST-RPE (e) layers after optimizing the wavefront for the IS/OS layer. (f-g) FFOCT en-face images acquired at the NFL and ganglion cell layer (GCL) boundary after optimizing the wavefront for the NFL. Images were acquired at 9^∘^ Nasal after correcting 3 Zernike modes. Magnified views (1–6) are presented below, highlighting different features detected by the FFOCT at various retinal depths.

To address this issue, we implemented the depth-resolved wavefront optimization strategy previously described in [15,18]. In this approach, only the brightness of the retinal layer of interest in the SD-OCT B-scan is considered as the merit function. Due to the large stroke of the DM, we can precisely align the focal plane with the FFOCT coherence gate position. This strategy enabled optimized SNR and resolution for imaging both the inner and outer retina, as demonstrated in Fig. 5. Figures 5 (a-c) show SD-OCT B-scans under three conditions: 1) before wavefront optimization; 2) after optimizing for the IS/OS layer; and 3) after optimizing for the NFL.

Following wavefront optimization at a target retinal layer, en-face FFOCT images can be acquired with optimized SNR and resolution across different retinal depths. Figures 5(d-g) present FFOCT images acquired at 9^∘^ Nasal from the same subject. When optimizing for the outer retina, the photoreceptor mosaic becomes clearly visible at IS/OS layer (Fig. 5(d)). Figure 5(e) illustrates an image acquired with a tilted coherence gate relative to the photoreceptor layer, allowing for the visualization of different retinal layers in a single frame. From left to right, the image reveals the photoreceptor mosaic at COST layer (Zoom 3 in Fig. 5), and the putative retinal pigment epithelium (RPE) (Zoom 4 in Fig. 5) with a characteristic speckle-like pattern when limited averaging is applied [39]. When optimizing the wavefront for the inner retina, the NFL can be visualized (Fig. 5(f)). Figure 5 demonstrates a deeper section at the boundary between the NFL (bottom of the image) and the ganglion cell layer (GCL, top of the image), revealing visible capillaries and characteristic GCL signal (Zoom 5 and 6 in Fig. 5(g)) without extensive averaging [40].

**Fig. 6.**
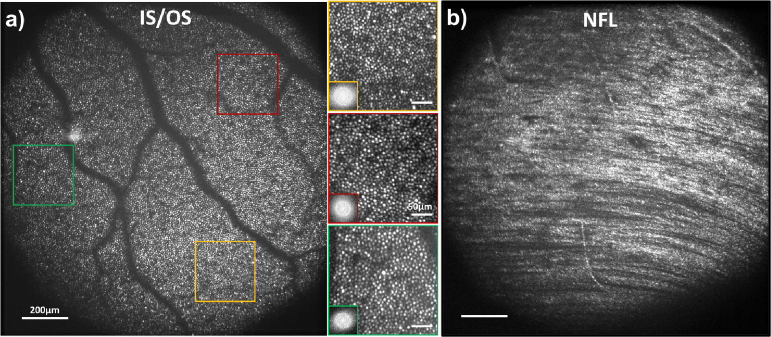
Depth-resolved FFOCT images of IS/OS layer (a) and NFL (b) at 4^∘^ superior and 2^∘^ nasal after 5-mode wavefront optimization.

Figures 6(a,b) present another example of a different subject, located at the 4^∘^ Superior and 2^∘^ Nasal eccentricity. In these images, individual photoreceptors and nerve fiber bundles are clearly distinguished, with enhanced SNR and resolution throughout $5^{\circ }\times 5^{\circ }$ FOV. Supplement 1 Figure S3 provides a third example from another subject and eccentricity, showcasing both photoreceptor and NFL imaging. In the latter, the vessel wall is clearly visible, providing an important structure for extracting biomarkers such as the wall-to-lumen ratio involved in many vascular diseases [41] and for studying more fundamental vascular functions, such as neurovascular coupling [42].

#### 3.3.3. Multi-depth imaging of the inner retina

**Fig. 7.**
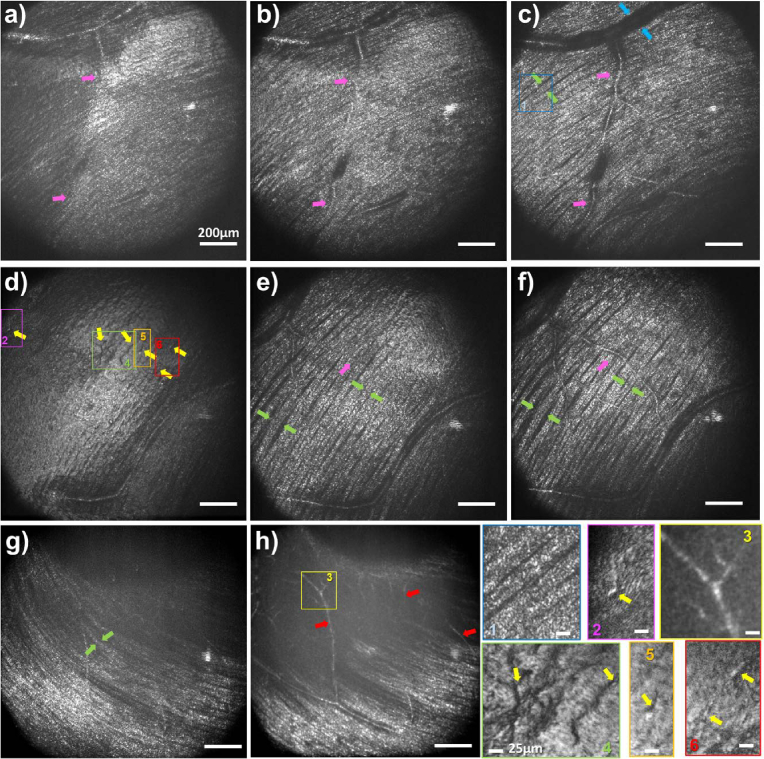
FFOCT images from the inner retina at different depths. (a-c) Images acquired at 6^∘^ superior and 2^∘^ temporal (d-f) Images acquired at 5^∘^ inferior and 7.5^∘^ temporal (g-h) Images acquired at 4^∘^ superior and 1^∘^ temporal. Arrows indicates different inner retinal features, such as vessels that go beneath the fibers (magenta arrows), vessel wall (blue arrows), putative macrophages (yellow arrows), individual nerve fiber bundles (green arrows), and capillaries (red arrows). Magnified images are presented on the bottom right, outlining some of these features.

As previously demonstrated, the Zernike mode-based sensorless AO approach using the large stroke of the DM improves both SNR and resolution in FFOCT, especially for inner retina imaging. This new capability, combined with an 8-*μm* FFOCT coherence gate, allows high-resolution imaging of inner retinal structures at different depths in a single acquisition. To achieve this, we simply introduce offset steps in the reference arm during acquisition. Figure 7 shows three examples captured at various depths within the inner retina, ranging from the inner limiting membrane (ILM) to the ganglion cell layer. This multi-depth imaging approach facilitates the visualization of fine inner retinal features over a large FOV, some of which are revealed for the first time when using time-domain FFOCT.

At the ILM level, Gunn’s dots can be observed (see Supplement 1 Figure S3) [43,44], along with the macrophage-like structures (yellow arrows in Fig. 7) positioned just above the ILM [45]. NFL imaging reveals additional features in Fig. 7, including individual fiber bundles (green arrows), and micron-sized hyper-reflective spots along the axon bundles (see magnified panel 1), whose size is consistent with axonal varicosities [46]. Notably, as the imaging depth slightly increases within the NFL, vessels progressively become more visible within the fiber bundles (magenta arrows). Finally, moving to the boundary between the NFL and GCL (Fig. 7(g-h)), capillaries (red arrows) begin to emerge. The vessel walls can also be visualized (blue arrows) in Fig. 7 (c)), as well as in Supplement 1 Figure S3 (blue arrows).

## 4. Discussion

In this manuscript, we proposed a DM-based sensorless AO time-domain FFOCT system for cellular-resolution retinal imaging over a large FOV. Using both phantom eye simulations and in vivo experiments, we demonstrated that correction of only three to five Zernike modes (defocus, astigmatism, and coma) was sufficient to enhance SNR and resolve fine retinal structures across a $5^{\circ }\times 5^{\circ }$ FOV (Figs. 4, 6). This approach enabled reliable visualization of foveal cones as close as 0.3^∘^ from the foveal center, as well as depth-resolved imaging of the photoreceptor at IS/OS and COST layers, and inner retinal features such as nerve fiber bundles, vessel walls, capillaries, internal limiting membrane, macrophage-like cells, and Gunn’s dots —- some of them for the first time using time-domain FFOCT. Compared with our previous implementation with the adaptive lens [18], the new design offers improved robustness across subjects with varying refractive errors, enhanced reproducibility of optimization, and the ability to image both the outer and inner retina in high resolution with improved SNR.

The main advance of this system over our earlier adaptive lens design [18,19] lies in the integration of a pupil-conjugated DM with a large stroke and negligible hysteresis. This configuration allows for precise matching of the coherence gate with the focal plane, which is particularly crucial for imaging the inner retina and for subjects with high refractive errors. The negligible hysteresis of the DM also enables a Zernike mode–based optimization strategy, resulting in faster convergence, more precise corrections, and minimal variability across repeated runs. In particular, both the HCL and DONE algorithms provided comparable performance with optimization times below 2 s, confirming the robustness of the approach. Together, these improvements translate into higher and more consistent SNR, particularly for inner retinal imaging, and greater reliability across a broader population compared to our previous adaptive-lens sensorless FFOCT system.

To achieve cellular resolution and visualize cones near the foveal center through a 7mm pupil, conventional AO ophthalmoscopes require sensor-based AO operating in real time to correct approximately 38 Zernike modes [32] and to account for dynamic ocular aberrations [22]. In contrast, sensorless AO systems, although attractive for their simplified and compact design, typically suffer from slower convergence relative to the dynamics of ocular aberrations and from the number of modes that must be corrected. This results in reduced AO performance and directly impacts the lateral resolution of the system. Consequently, no conventional sensorless ophthalmoscope (for example OCT, SLO, or FIO) has yet achieved diffraction-limited imaging with a 7-mm pupil to resolve the smallest densely packed foveal cones [11–15,47,48].

We believe that our ability to achieve such high resolution with a sensorless AO approach relies on the unique properties of time-domain FFOCT. Indeed, the use of spatially incoherent illumination in FFOCT leads to a lateral resolution that is twice that of conventional ophthalmoscopes [20]. In addition, it demonstrates greater resistance against dominant ocular aberrations, such as defocus and astigmatism [20,21]. Consequently, FFOCT can attain cellular resolution with a less complex aberration correction strategy and a reduced number of corrected modes [19,21]. Moreover, because dynamic ocular aberrations are dominated by defocus and astigmatism [22], we believe that time-domain FFOCT is expected to maintain high resolution following sensorless AO correction for acquisitions of 3 seconds [21]. Regarding the high resolution observed across the full $5^{\circ }\times 5^{\circ }$ FOV, this cannot be attributed to the merit function, since it only considers a narrow retinal region (1^∘^ SD-OCT B-scan at the same location). Instead, we attribute this wide-field performance to the fact that field-dependent ocular aberrations are also dominated by low-order modes, primarily defocus and astigmatism [10]. Therefore, the current $5^{\circ }\times 5^{\circ }$ FOV is determined by the number of pixels on the detector and the chosen spatial sampling (1*μ*m per pixel in the retinal plane to resolve 2*μ*m features). A larger FOV could, in principle, be achieved by employing a detector containing more pixels or by relaxing the spatial sampling, at the expense of lateral resolution. Finally, in FFOCT, the main impact of ocular aberrations is a reduction in the SNR, as aberrated photons do not interfere effectively. Therefore, a critical step in this sensorless AO design was to determine the optimal number of Zernike modes for correction in order to maximize SNR. To this end, we used a phantom eye protocol in which ocular aberrations were simulated by directly adding them to the DM while imaging a scattering sample.

Using this protocol, we found that for approximately two-thirds of the population studied (HOA groups 1 and 2), correction of only three Zernike modes (defocus and astigmatism) was sufficient to optimize the FFOCT SNR (Fig. 2, 3). For eyes with stronger high-order aberrations (group 3), extending the correction to five modes (up to coma) was necessary to optimize SNR and retrieve high-resolution images (Fig. 2, 3). This minimal correction enabled reliable visualization of foveal cones as close as 0.3^∘^ from the foveal center across a $5^{\circ }\times 5^{\circ }$ FOV at 500 Hz and inner retinal features, without apparent anisoplanatism (Fig. 4).

Another contribution of this study is the use of the phantom eye protocol to define the optimal number of Zernike modes required for sensorless AO in FFOCT. Beyond this specific application, the protocol is versatile and could be applied to other ophthalmoscopes or microscopy modalities. In these contexts, it may serve as a practical tool not only to determine the number of modes to be corrected, but also to refine other critical parameters of sensorless AO algorithms (such as the number of cycles, iterations, or hyperparameters) which are often selected empirically. In doing so, the phantom eye protocol offers a systematic way to benchmark and optimize sensorless AO designs.

Despite its advantages, the current implementation of DM-based sensorless AO time-domain FFOCT presents some limitations. The primary challenge is its relatively low sensitivity, which restricts its ability to distinguish weakly reflective retinal features, such as ganglion cells. This low sensitivity can be attributed to three factors. First, time-domain OCT inherently possesses a lower SNR compared to Fourier-domain OCT systems [49]. Second, the FFOCT camera detects multiply scattered photons, which increases noise and decreases overall SNR. Third, although the sensorless AO approach significantly improves SNR and is sufficient to achieve cellular resolution, dynamic aberrations and uncorrected high-order static aberrations prevent the final SNR from reaching the diffraction limit. In the case of uncorrected high-order aberrations, this effect on SNR can be seen in Fig. 2, where the SNR reaches 40 dB, 37 dB, and 35 dB for HOA groups 1, 2, and 3, respectively, compared to 45 dB at the diffraction limit. Concerning dynamic aberrations, we have shown in a previous publication that a loss in SNR is expected to be around 3dB for groups 1 and 2, and 10dB for group 3 when using the sensorless approach [21]. This SNR deficit could be mitigated by implementing a sensor-based AO strategy to further boost the FFOCT SNR [21], although this would increase the complexity of the system. Alternatively, the relatively low sensitivity of FFOCT could be addressed through modified illumination and detection schemes to filter out multiply scattered photons [50–54]. A further limitation is the increased system footprint due to the DM pupil conjugation. Given that a three-mode correction is sufficient for a relatively large proportion of the population (approximately two-thirds), it would be possible to adapt our previous adaptive-lens approach by combining two adaptive lenses in a woofer–tweeter configuration. In such a design, a variable-focus lens would correct defocus with a large stroke, while a second adaptive lens would correct astigmatism with a smaller stroke [55].

## 5. Conclusion

In conclusion, this work demonstrates that DM-based sensorless AO can be successfully combined with time-domain FFOCT to achieve wide-field, cellular-resolution retinal imaging with minimal modal correction. Our results, validated through both phantom eye protocol and in vivo experiments, show that correcting for less than five Zernike modes is sufficient to achieve high SNR and resolve fine retinal structures such as foveal cones and inner retinal features. Although challenges remain, particularly in achieving diffraction-limited SNR for less reflective structures, our approach establishes sensorless AO FFOCT as a practical and scalable solution for high-resolution retinal imaging and offers a promising pathway toward clinical deployment. Future improvements in sensitivity, algorithmic optimization, and compact optical designs are expected to further enhance performance and extend applicability to a broader range of patient populations and disease contexts.

## Supplemental information

Supplement 1 Supplemental Document https://doi.org/10.6084/m9.figshare.30542426

Visualization 1 Wavefront optimization process using a sensorless AO approach with the brightness of the SD-OCT signal as a merit function. SD-OCT B-scan (left panel), lateral average of the SD-OCT B-scan (upper right panel) and merit function during the AO sensorle https://doi.org/10.6084/m9.figshare.30156499

## Data Availability

Data underlying the results presented in this paper are not publicly available at this time but may be obtained from the authors upon reasonable request.

## Supplemental document

See Supplement 1 for supporting content.
