# Supplementary material for: Wide-field cellular-resolution retinal imaging using deformable mirror-based sensorless adaptive optics time-domain full-field OCT
Source: Biomed Opt Express. 2025 Nov 17;16(12):5179–96. doi: 10.1364/BOE.579540 (PMC12698103; doi:10.1364/BOE.579540)
Supplement: Supplementary file 1 [file boe-16-12-5179-s001.pdf]

Supplemental Document

Biomedical Optics EXPRESS

Wide-field cellular-resolution retinal imaging using deformable mirror-based sensorless adaptive optics time-domain full-field OCT: supplement

YAO CAI,[1,2] OLIVIER MARTINACHE,[1] MAXIME BERTRAND,[3] CLÉMENTINE CALLET,[2] OLIVIER THOUVENIN,[1] KATE GRIEVE,[2] AND PEDRO MECÊ[1,*]

[1] *Institut Langevin, ESPCI Paris, CNRS, PSL University, Paris, France*
[2] *Sorbonne Université, INSERM, CNRS, Institut de la Vision, Paris, France*
[3] *SharpEye SAS, Gentilly, France*
[*] *pedro.mece@espci.fr*

This supplement published with Optica Publishing Group on 17 November 2025 by The Authors under the terms of the Creative Commons Attribution 4.0 License in the format provided by the authors and unedited. Further distribution of this work must maintain attribution to the author(s) and the published article's title, journal citation, and DOI.

Supplement DOI: https://doi.org/10.6084/m9.figshare.30542426

Parent Article DOI: https://doi.org/10.1364/BOE.579540

1. USING THE SD-OCT SIGNAL AS A MERIT FUNCTION

As FFOCT images suffers from intensity fluctuation arising from inaccurate two-phase modulation induced by the retinal axial motion, we decided to use the brightness of the SD-OCT B-scan as a surrogate for FFOCT SNR optimization. Given the close wavelengths of both systems, wavefront optimization using SD-OCT brightness as the merit function effectively enhances the FFOCT SNR as well (Fig. S1).

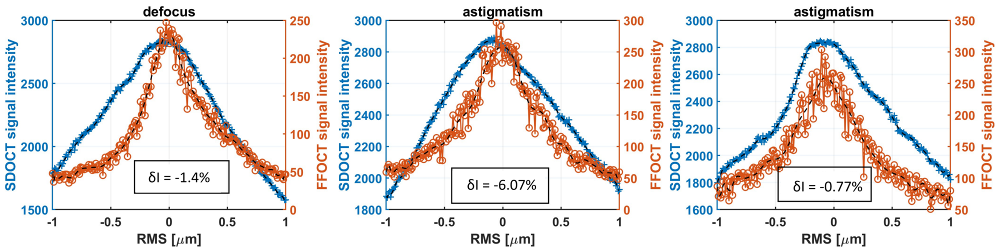

Fig. S1. SDOCT signal (blue) and FFOCT signal (orange) as a function of various Zernike coefficients for defocus (left panel), and both astigmatisms (central and right panels).

The optimal Zernike coefficients exhibited only a small difference between SD-OCT and FFOCT, with reductions in FFOCT signal intensity of 1.4%, 6%, and 0.77% when applying the coefficients optimized for SD-OCT to correct defocus and the two astigmatic components, respectively. This difference can be due to chromatic aberrations, as wavelgths are slighly different or non-common path aberrations.

2. PHOTORECEPTORS IMAGING OVER A LARGE FIELD-OF-VIEW

Figure S2 presents four photoreceptor images acquired at IS/OS level for different subjects and eccentricities. Magnified images highlight that cones are visible throughout the whole field-of-view, without any apparent anisoplanatism.

3. MULTI-DEPTH INNER RETINAL IMAGING

We provide in Fig.S3 another example of multi-depth inner retinal imaging for the same subject at slightly different Nasal eccentricities. In particular, one can notice that as the imaging depth slightly increases within the NFL, vessels progressively become more visible within the fiber bundles (magenta arrows). At ILM depth, Gun's dot are visible (yellow arrows). As we approach the ganglion cells layer, vessel wall (blue arrows) and capillaries become visible.

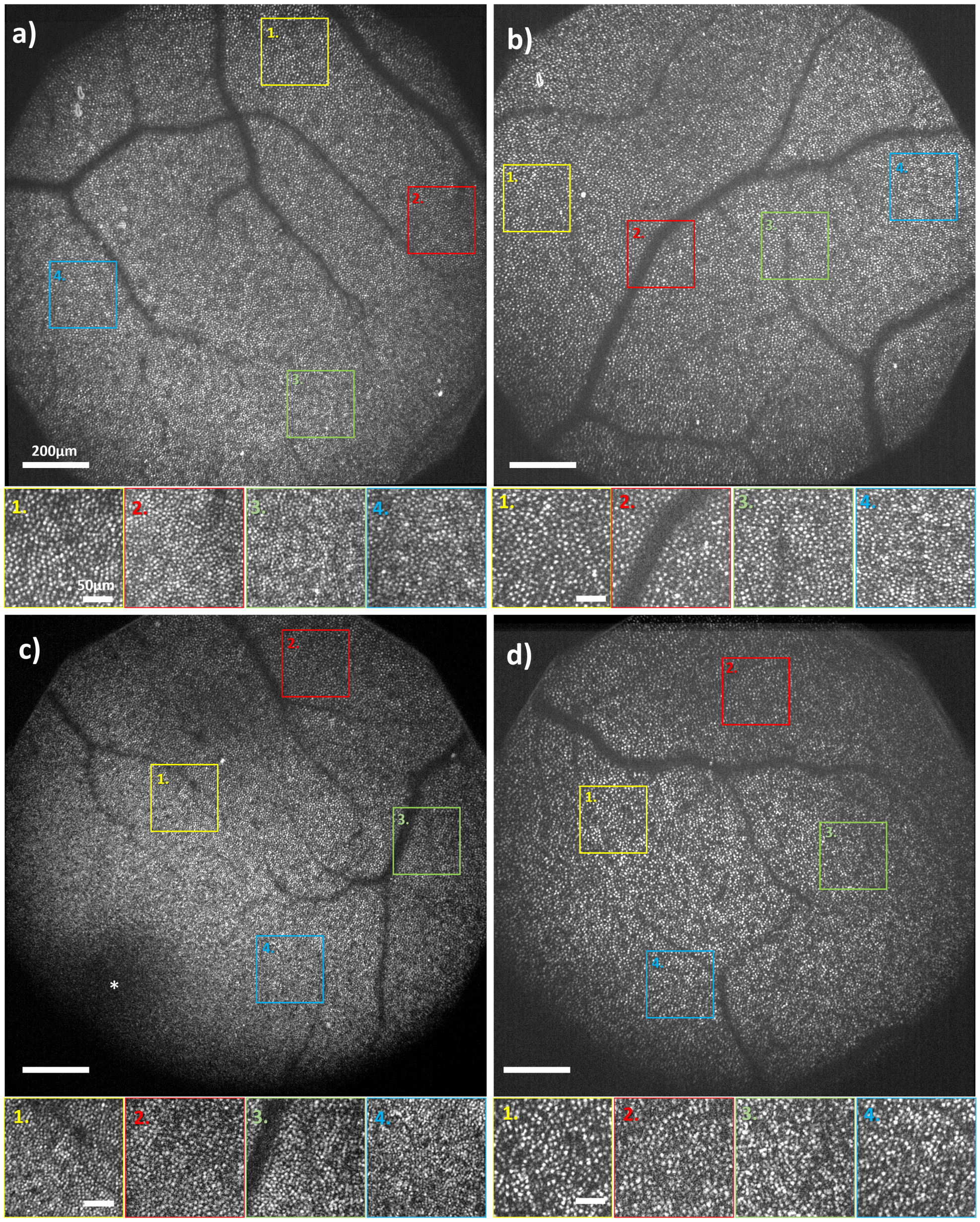

Fig. S2. FFOCT en-face images of photoreceptors IS/OS for 4 different subjects and eccentricities. a) 2° Superior and 3° Nasal. b) 1° Inferior and 6° Nasal. c) Near the foveal center (indicated by *). d) 7° Nasal.

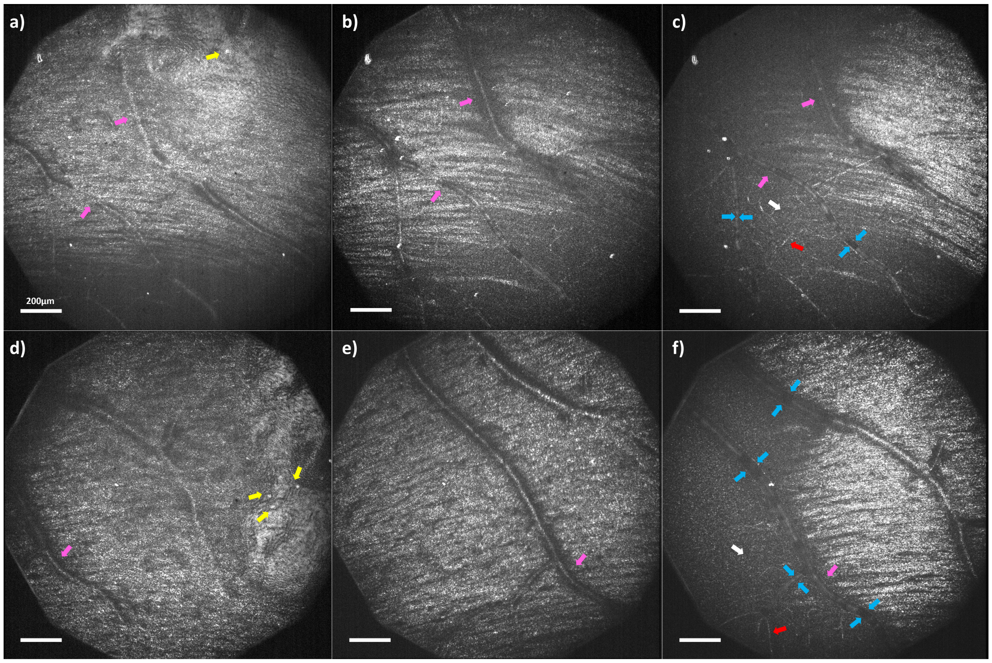

Fig. S3. (a-c) and (d-f) two examples of multi-depth inner retina imaging going from the ILM to the ganglion cell layer.
